# Establishing an Expert Consensus on Key Indicators of the Quality of Life among Breast Cancer Survivors: A Modified Delphi Study

**DOI:** 10.3390/jcm11072041

**Published:** 2022-04-05

**Authors:** Izidor Mlakar, Simon Lin, Jama Nateqi, Stefanie Gruarin, Lorena Diéguez, Paulina Piairo, Liliana R. Pires, Sara Tement, Ilona Aleksandraviča, Mārcis Leja, Krista Arcimoviča, Valérie Bleret, Jean-François Kaux, Philippe Kolh, Didier Maquet, Jesús Garcia Gómez, Jesus García Mata, Mercedes Salgado, Matej Horvat, Maja Ravnik, Vojko Flis, Urška Smrke

**Affiliations:** 1 Faculty of Electrical Engineering and Computer Science, University of Maribor, 2000 Maribor, Slovenia; urska.smrke@um.si; 2 Data Science Department, Symptoma, 1030 Vienna, Austria; lin@symptoma.com; 3 Medical Department, Symptoma, 4864 Attersee, Austria; nateqi@symptoma.com (J.N.); gruarin@symptoma.com (S.G.); 4 RUBYnanomed, 4700-314 Braga, Portugal; lorena.dieguez@rubynanomed.com (L.D.); paulina.piairo@rubynanomed.com (P.P.); liliana.pires@rubynanomed.com (L.R.P.); 5 Faculty of Arts, Department of Psychology, University of Maribor, 2000 Maribor, Slovenia; sara.tement@um.si; 6 Institute of Clinical and Preventive Medicine of the University of Latvia, LV-1586 Riga, Latvia; ilona.aleksandravica@lu.lv (I.A.); marcis.leja@lu.lv (M.L.); 7 Oncology Centre of Latvia, Riga East Clinical University Hospital, LV-1038 Riga, Latvia; krista.arcimovica@gmail.com; 8 Department of Senology, University Hospital of Liège, 4000 Liège, Belgium; valerie.bleret@matble.be (V.B.); d.maquet@uliege.be (D.M.); 9 Physical and Rehabilitation Medicine, University Hospital of Liège, 4000 Liège, Belgium; jfkaux@ulg.ac.be; 10 Department of Information Systems Management, University Hospital of Liège, 4000 Liège, Belgium; philippe.kolh@chuliege.be; 11 Oncology Department, University Hospital Complex of Ourense (SERGAS), 32005 Ourense, Spain; jesus.garcia.gomez@sergas.es (J.G.G.); jesus.garcia.mata@sergas.es (J.G.M.); 12 Department of Medical Oncology, Galician Health Services (SERGAS), 15703 Santiago de Compostela, A Coruña, Spain; mercedes.salgado.fernandez@sergas.es; 13 Department of Oncology, University Medical Centre Maribor, 2000 Maribor, Slovenia; matej.horvat@ukc-mb.si (M.H.); maja.ravnik@ukc-mb.si (M.R.); vojkoflis@gmail.com (V.F.)

**Keywords:** breast cancer survivorship, health-related quality of life, Delphi study, guidance

## Abstract

(1) Background: The needs of cancer survivors are often not reflected in practice. One of the main barriers of the use of patient-reported outcomes is associated with data collection and the interpretation of patient-reported outcomes (PROs) due to a multitude of instruments and measuring approaches. The aim of the study was to establish an expert consensus on the relevance and key indicators of quality of life in the clinical practice of breast cancer survivors. (2) Methods: Potential indicators of the quality of life of breast cancer survivors were extracted from the established quality of life models, depicting survivors’ perspectives. The specific domains and subdomains of quality of life were evaluated in a two-stage online Delphi process, including an international and multidisciplinary panel of experts. (3) Results: The first round of the Delphi process was completed by 57 and the second by 37 participants. A consensus was reached for the Physical and Psychological domains, and on eleven subdomains of quality of life. The results were further supported by the additional ranking of importance of the subdomains in the second round. (4) Conclusions: The current findings can serve to optimize the use of instruments and address the challenges related to data collection and interpretation as the facilitators of the adaption in routine practice.

## 1. Background

The long-term effects of breast cancer and its treatment have been shown to have both positive and negative effects on the recovery and quality of life (QoL) of survivors [1,2], exceeding only physical effects. In developed countries, the mortality rate has declined significantly in recent years, due to improved screening, early diagnosis, and treatment programs [3,4]. Most patients seem to adjust well; however, over time, cancer survivors often start to suffer from decreased well-being [5,6]. The need to focus more on the QoL of cancer survivors is recognized by the EU Beating Cancer Plan [7], which supports the shift in focus from “‘how long’ people live after diagnosis, rather to ‘how well and how long’ they live” [6] (p. 21), and is recognized as one of the most important intervention areas to focus on by the European Commission’s Mission Board for Cancer [8].

QoL represents an essential end point in cancer survivorship, arguably as important as the therapy response, disease-free, and overall survival rates [9]. While there are a multitude of QoL definitions and models that, in focus, range from including only specific domains of QoL to approaches that emphasize the impact of cancer on persons’ health, it is clear that the definitions of the QoL of cancer survivors tend to be separated from those of cancer patients [10]. Ferrell et al. [11,12] developed a model of QoL specific to cancer survivors, which has been employed widely and consistently in the last two and a half decades. Even though since the first publication of the model, several variations and adaptations on it have been published [1,10,13,14], the four general domains are recognized consistently; i.e., the Physical, Psychological, Social, and Spiritual domains. Within the concept of four domains and subdomains, a multitude of questionnaires and patient-reported outcomes (PROs) were designed, for monitoring the well-being of breast and other cancer patients and survivors. The most widely used, such as the European Organization for Research and Treatment of Cancer core questionnaire (EORTC QLQ-C30) [15,16,17], Functional Assessment of Cancer Therapy—General (FACT-G) [18], and the BREAST—Q Patient-Reported Outcomes Instrument [19], focus predominantly on clinical over psychological factors. Other questionnaires, such as Cancer Problems in Living Scale (CPILS) [20], the multidimensional instrument measuring cancer-related fatigue (EORTC QLQ-FA12) [21], Quality of Life in Adult Cancer Survivors (QLACS) [22], and Quality of Life Cancer Survivors (QoL-CS) [11], have been developed to focus on psychosocial aspects and fatigue related to survivorship. These, however, do not include clinical or bodily/corporal condition-specific issues, and only limited information has been reported on their psychometric properties [23].

Patient-reported outcomes (PROs) are particularly important, since these tools can capture a patient’s subjective perception of the effect of their disease and treatment on the physical, psychological and social aspects of daily life [24] and can lead to person-centered survivorship, with a strong focus on prevention, early detection of late effects and timely intervention when health risks/conditions occur [25]. While individual questionnaires aim to capture the important QoL domains, in practice, where a global vision of a survivor would be favorable, an application of a multitude of these questionnaires to enable consideration of their targeted nuances of their overlapping domains is not feasible. Strongly differing approaches to the measurement of cancer patients’ and survivors’ well-being may also reflect a limited consensus of which domain(s) of the QoL should be monitored in detail. While little is known regarding the QoL of long-term breast cancer survivors, the issues resulting from breast cancer and its treatment are varied and complex, and those faced by survivors differ from the ones experienced by patients [26]. One of the main barriers to integrate PROs in clinical practice includes professionals’ lack of time and knowledge to interpret and integrate PRO data meaningfully into practice, and the inability for PRO data to be acted upon [27]. Furthermore, the issues are often explored in survivors living in the United States, limiting their generalizability to survivors in other countries [1]. Moreover, most of the existing questionnaires focus on the Physical and Psychological domains. However, the perspective of patients clearly highlights the importance of the four domains of survivor’s well-being, namely, Psychological, Social, Physical, and Spiritual [1,28]. Social and spiritual distress, including fertility preservation and family planning, are crucial issues yet to be addressed comprehensively by practice in all cancer patients and survivors, especially those of reproductive age [29,30,31].

The primary aim of this study is to establish an expert consensus on the key indicators of the QoL of breast cancer survivors from HCPs’ perspectives. The goal is not to replace existing tools, but to provide a consensus on the clinical importance of the domains and subdomains of QoL in the routine of breast cancer survivorship. This could, in turn, enable refining of the nature of existing, but especially, future questionnaires, and improve their integration by aligning them further with clinical practice. The already established domains and subdomains of QoL that depict cancer survivors’ perspectives were evaluated by healthcare professionals (HCPs), regarding their importance in the follow-up and monitoring of breast cancer survivors through a modified two-stage Delphi study. In order to aid to the generalizability of the findings in relation to the fields of expertise and healthcare systems and settings in the EU area, experts of various backgrounds involved in cancer care of long-term breast cancer survivors were involved from seven European countries.

## 2. Methods

The methodology of the present study consists of a modified Delphi procedure, adapted from related studies [32]. Similar to Tung et al. [33], a two-step strategy was applied to identify the most important domains and subdomains of QoL monitoring in the care of breast cancer survivors. First, a pool of potentially important subdomains of QoL was identified following the existing models of QoL applied to cancer patients and survivors. Second, an international multidisciplinary panel of experts evaluated these items in a two-round Delphi process, reaching a consensus on the most important subdomains of QoL.

### 2.1. QoL Domain and Subdomain Identification

A model of QoL in cancer survivors by Ferrell et al. [11] was chosen as a theoretical basis of the QoL domains and subdomains in this study. As several variations and adaptations, especially of its subdomains, exist, the first step of the study was the mapping of the subdomains among the variations of the model by Ferrell et al. [10,11,13,14], which enabled the identification of overlapping subdomains, as well as specific ones. The extracted subdomains were reviewed by the authors, and reformulated when needed to reach greater clarity, in order to represent an extensive list of clearly defined QoL subdomains. The resulting list was reviewed by HCPs and the researchers in the research team. Two additional subdomains, often observed in practice by the HCPs involved, were added (health distress in physical, and loss of interest in usual activities in the Psychological QoL domain), arriving at four general QoL domains (i.e., Physical, Psychological, Social, and Spiritual), and 35 subdomains, which formed the questionnaire items evaluated in Round 1 of the Delphi process.

### 2.2. QoL Domain and Subdomain Identification

The initial, preparatory phase of a Delphi process often consists of a qualitative, or a mix of qualitative and quantitative surveys, where experts are encouraged to provide insights into the researched topic [34,35]. In this case, the preparatory phase was carried out following a customizable approach [36]. As such, 8 researchers, clinical experts of the project Patients-centered SurvivorShIp care plan after Cancer treatments based on Big Data and Artificial Intelligence technologies (PERSIST) [37] carried out a review of the QoL models. The final questionnaire on the subdomains of QoL to be evaluated in the next phases was formulated based on an online discussion between the experts of the project PERSIST. In phase 2 an online survey was designed, evaluating the importance of the identified subdomains of the QoL, and conducted in two rounds. The two rounds were conducted in spring and fall 2020. In both rounds, potential participants received an invitation by e-mail explaining the study and a link to the online questionnaire. To be followed through both rounds, participants were given a unique ID at the beginning of the first round. Informed consent was sought at the beginning of each round. All procedures in this study were performed in accordance with the ethical standards of the Institutional and National Research Committees, and with the Helsinki Declaration and its amendments. A quantitative analysis was carried out in phase 3. The results of the analysis are reported in Section 3 of this research.

This study is part of a clinical study carried out under the project PERSIST [37], for which ethical approvals were obtained from the Institutional Ethics Committee of CHU de Liège (Approval Ref. No: 2020/248), Riga Eastern Clinical University Hospital Support Foundation Medical and Biomedical Research Ethics Committee (Approval Ref. No: 8-A/20), the National Ethics Committee, Ministry of Health, Slovenia (Approval Ref. No. 0120-352/2020/5), and the Pontevedra-Vigo-Ourense Research Ethics Committee (Approval Ref. No. 2020/394).

#### 2.2.1. Participants

HCPs involved in the follow-up of breast cancer survivors were recruited for this study by an adapted method of Borgiel and colleagues [33,38]. Members of the research team acted as recruiters and contacted their professional peers, informing them about the study and inviting them to participate. Special emphasis was put on recruiting participants from various areas of expertise (e.g., Oncologists, Nurses, Psychologists, Physiotherapists). Since the healthcare systems and care paths for cancer patients and survivors can differ substantially between different countries, participants were recruited from seven European countries (i.e., Belgium, Italy, Latvia, Portugal, Slovenia, Spain, and Switzerland).

Altogether, 85 HCPs participated in the study. The Delphi method usually requires the inclusion of most of the experts in all survey rounds of the study. In the present study, the participation of experts involved in the first survey round was hindered significantly during the second quantitative round, due to the unavailability of many professionals during the COVID-19 pandemic. Therefore, only 15.8% of the participants in the first round were included in the second round of the study. The recruitment process of round 1 (email invitations) was repeated to obtain generalizable results. The impact of the low overlap between the first and second quantitative rounds is discussed further under Study Limitations.

#### 2.2.2. Questionnaire

The questionnaire for the first round consisted of 39 subdomains identified in the review of existing QoL models, i.e., the variations of the model by Ferrell et al. [10,11,13,14] (see Section 2.1). Participants were asked to rate their importance in the follow-up of the breast cancer survivors on a 7-point scale (1—not important; 7—very important). For the second-round questionnaire, subdomains were retained that reached the consensus criterion (see Section 2.2.3) in the first round. Participants were provided feedback in the form of the median answer and percentage of participants giving that answer in the first round for each subdomain. Participants rated the importance for the retained subdomains again on the 7-point scale. In the second round, participants were additionally asked to rank the subdomains in order of importance.

#### 2.2.3. Consensus Criterion and Analyses

While there is no generally accepted rule for establishing the criterion for consensus in Delphi studies, one of the most common approaches is setting a specific percentage level of agreement, which varies from 51 to 100% [34,35]. This approach was also followed in the present study, setting the criterion at 75% (similar to [39,40]).

In the first round, consensus for a subdomain was reached if at least 75% of participants evaluated it within the top three scores of a 7-point scale. A stricter criterion was applied in the second round. The consensus was reached if at least 75% of the participants evaluated a subdomain within the top two scores of the 7-point scale.

Intraclass correlation coefficients (ICC) based on a mean-rating 2-way mixed-effects model were calculated, to assess the consistency of the raters for each round. Since the second round included two groups of participants, namely, the experts who were already included in the first round and additional ones included only in the second round, *t*-tests were calculated for assessing the possible differences between the judgments of these two groups. Analyses were performed using R version 4.0.3 [41] (and the packages psych [42] and rstatix [43]).

## 3. Results

### 3.1. Round 1

The questionnaire for Round 1 of the study was completed by 57 experts (see Table 1). The results of interrater reliability analysis indicated good to excellent reliability [44] of the raters, with ICC (2, 57) = 0.90, 95% CI (0.86, 0.93).

The consensus was reached for three of the four general domains (75.00%; see Table 2), namely, Physical, Psychological, and Social QoL, and 30 of the 35 subdomains (85.71%): In the Physical and Psychological QoL domains, all the subdomains reached a consensus (i.e., 10, 100.00% for both domains). In the domain of Social QoL, 8 out of 10 subdomains (80.00%), and in the Spiritual QoL domain, 2 out of 5 (40.00%) subdomains reached a consensus.

### 3.2. Round 2

The questionnaire for Round 2 was completed by 9 participants (15.79%) who participated in Round 1 (Group 1), and an additional 28 participants (Group 2; see Table 1), resulting in a total of 37 participants with good reliability of their ratings [44], ICC (2, 37) = 0.85, 95% CI [0.78, 0.91]. Given that independent samples *t*-tests indicated (see Table 2) that the two groups did not differ significantly in their mean evaluations of the QoL subdomains, it was concluded that the results of Round 2 reflected the opinion of both groups.

The consensus was reached for two out of three general domains (66.67%), namely, Physical and Psychological QoL. The consensus reached on the level of subdomains, 11 of 30 (36.67%), were as follows: Physical QoL 7 out of 10 (70.00%), Psychological QoL 2 out of 10 (20.00%), Social QoL 1 out of 8 (12.50%), and Spiritual QoL 1 of 2 (50.00%). In Round 2, participants were also asked to rank the importance of the subdomains (see Table 3).

The questionnaire for Round 2 was completed by 9 participants (15.79%) who participated in Round 1 (Group 1), and an additional 28 participants (Group 2; see Table 1), resulting in a total of 37 participants with good reliability of their ratings [44], ICC (2, 37) = 0.85, 95% CI [0.78, 0.91]. Given that independent samples *t*-tests indicated (see Table 2) that the two groups did not differ significantly in their mean evaluations of the QoL subdomains, it was concluded that the results of Round 2 reflected the opinion of both groups.

## 4. Discussion

Breast cancer survivors have complex and specific needs, often not reflected in clinical practice. The use of PROs in clinical practice has the potential to mitigate this issue and promote patient-centered, personalized care. There are a multitude of PROs measuring QoL and patient experiences available; however, only limited consensus has been reported on the subdomains of QoL to be monitored. In practice this is reflected through a slow uptake of PROs in routine practice, due to issues related to data collection (i.e., multiple instruments to be filled in by participants), data interpretation, and regulatory challenges due to ubiquitous understanding of patient experiences. Further, the current QoL questionnaires focus mostly on the Physical and Psychological domains, and mostly disregard the Social and Spiritual domains, deemed important by survivors themselves. Therefore, the present study aimed to establish an expert consensus on the key indicators of QoL subdomains, among breast cancer survivors, to constitute person-centered breast cancer survivorship.

Only a part of the QoL subdomains was recognized as important for monitoring breast cancer survivors from the HCPs’ perspectives. After two rounds of the study, two out of four general domains (i.e., Physical and Psychological QoL) proved to be important from the perspective of HCPs in the monitoring of breast cancer survivors, suggesting a predominant focus on two domains in daily practice. However, on the level of subdomains, the consensus was reached for several of them—not only within the Physical and Psychological QoL, but also within the domains of Social and Spiritual QoL.

These results were mostly supported by mean ranks of the importance of subdomains from the second round, i.e., on the level of general domains, Physical, Psychological, and Spiritual QoL, where, in general, subdomains that reached a consensus also reached lower mean ranks scores, implying higher importance. Some discrepancies between ranking and consensus results were observed in the subdomains of Psychological QoL, where the anxiety subdomain did not reach a consensus but surpassed, in importance, the depression and psychological distress subdomains, for which a consensus was reached. Similarly, within the Social QoL domain, the subdomain of role limitations (the only one that reached a consensus), was surpassed in importance ranking by five other subdomains. Such discrepancies may reflect the aforementioned predominant focus on Physical QoL in clinical practice—with less focus on other areas, the experts’ opinion might not be as elaborated as for the Physical QoL, and could, therefore, be more sensitive to the method of reporting, or be formed in the process of participation in the survey. A general consensus on importance but missing consensus on specific subdomains might also reflect differences in the value systems of cultures, religions, social groups, etc., but also a lack of objective instruments to account for these differences.

Nevertheless, there are several tools available for assessing the QoL of breast cancer patients and survivors (for review, see [1]), but none were found that would somewhat specifically reflect the results of our study. Being breast cancer specific and on the level of general domains assessing the physical and psychological (and additionally, social) aspects of the QoL, the Functional Assessment of Cancer Therapy-Breast (FACT-B) [46] appears to be reasonably aligned with the key subdomains identified in this study (with some important differences, for instance, regarding the Spiritual subdomains). Further, also specific for breast cancer survivors is the European Organization for Research and Treatment of Cancer-Breast Module (EORTC QLQ-BR23) [47], but it focuses mainly only on the Physical domain of the QoL [1]. Other identified questionnaires are more general in the target population or specific to other cancer types and vary substantially in the subdomains covered.

To measure the key indicators of the QoL of breast cancer survivors effectively, further studies are needed; that is, on the level of consensus on the indicators between HCPs and survivors, and also on the level of questionnaires, reflecting these indicators. Monitoring in the role of supporting the QoL of breast cancer survivors can be improved with the established consensus and corresponding questionnaires.

### Study Limitations

First, the low overlap of participants in both rounds represents a deviation from the standard Delphi methodology [34,35]. However, as Tung et al. [33] pointed out, the Delphi procedure is, to some extent, biased, as “the judgments of this expert panel may not be representative of all experts who were qualified to participate” [33] (p. 1225). Therefore, the inclusion of additional participants in the second round can help achieve a higher level of generalizability, which is also supported by the multidisciplinary and international make-up of the expert panel.

Second, the experts evaluated an already defined list of QoL subdomains, potentially limiting their own contributions to the topic. However, as Pietersma et al. [32] stated for a similar modification, evaluating a predefined set of domains can be less demanding cognitively, and it ensures that not only those domains are included that participants can think of easily. Additionally, since the included subdomains are based on QoL models for breast cancer patients, this represents a high probability that a sufficiently extensive pool of subdomains was included. Although the present study evaluates models of QoL, designed for and in collaboration with, breast cancer patients, the study did not involve patients directly. It reflects a subjective perception from the HCPs’ perspectives.

Third, age and the length of survivorship represent important confounders in further ranking on the importance of the subdomains of the QoL model. Namely, early survivorship represents a time-critical period, during which survivors of breast cancer attempt to resume functional activities and important life roles [48]. During this period, self-image, fatigue and the side effects of medication represent a significant burden, whereas, for long-term survivors, the highest unmet needs relate to the Health System and Information domain [49]. Furthermore, young cancer survivors are often burdened with fertility preservation and family planning [29], whereas older cancer survivors are faced with age-related declines in functioning, comorbid illnesses, and diminished social and economic resources [50,51].This limitation should be addressed in future studies.

## 5. Conclusions

The current Delphi study exploited the already established perspectives of breast cancer survivors on QoL, and through a two-round process, reached an expert consensus on the most important subdomains for the monitoring of breast cancer survivors. The goal of this study was to identify the key indicators of the QoL of breast cancer survivors from the experts’ perspectives, i.e., those that have an important impact on the design/delivery of the survivorship pathway. The results show clearly that consensus was reached on the Physical and Psychological domains, and on 11 of 30 subdomains. This study may serve as the baseline towards optimization and standardization of instruments to be used in the routine monitoring of breast cancer survivorship. The study clearly supports the notion that there is a certain level of mismatch between expert perception of what is important and the key indicators of QoL identified by patients. This study further highlights the importance of including both HCPs and patients when setting research priorities, and in designing a patient-centered approach to cancer survivorship care. Further studies, however, are needed, to establish a way for these findings to truly enhance the support breast cancer survivors receive. To truly appreciate the subjective perception of a patient, a comparative study with cancer patients and survivors should be carried out. In this way ‘a lack of value’ may be prevented, which is often perceived by patients [27]. Furthermore, differences in personal value systems of breast cancer survivors, depending both on their age and on the duration of survivorship, should be analyzed and reflected in further refinements of patient-centric QoL models.

## Figures and Tables

**Table 1:** Participants’ characteristics. Group 1: Participants included in both rounds. Group 2: Participants included only in the 2nd round.

		Round 1		Round 2		Group 1		Group 2	
				All Participants					
		f	%	f	%	f	%	f	%
N		57		37		9		28	
Gender	Female	40	70.18	27	72.97	7	77.78	20	71.43
	Male	17	29.82	10	27.03	2	22.22	8	28.57
Specialty	Gynecology	1	1.75	-	-	-	-	-	-
	Kinesitherapy	1	1.75	-	-	-	-	-	-
	Medical oncology	15	26.32	10	27.03	3	33.33	7	25.00
	Nuclear medicine	2	3.51	-	-	-	-	-	-
	Nursing	2	3.51	-	-	-	-	-	-
	Oncology nursing	7	12.28	1	2.70	-	-	1	3.57
	(Oncology) Research	2	3.51	-	-	-	-	-	
	Pathology	1	1.75	-	-	-	-	-	-
	Pharmacy	4	7.02	-	-	-	-	-	-
	Physiotherapy	1	1.75	5	13.51	-	-	5	17.86
	Psychiatry	1	1.75	-	-	-	-	-	-
	Psychology	5	8.77	3	8.11	-	-	3	10.71
	Psychotherapy	2	3.51	-	-	-	-	-	-
	Radiology	3	5.26	2	5.41	-	-	2	7.14
	Radiotherapy	6	10.53	7	18.92	3	33.33	4	14.29
	Surgery	5	8.77	5	13.51	1	11.11	4	14.29
	Teacher	1	1.75	-	-	-	-	-	-
	Volunteer	1	1.75	-	-	-	-	-	-
	Other	16	28.07	4	10.81	2	22.22	2	7.14
Country	Belgium	15	26.32	17	45.95	4	44.44	13	46.43
	Italy	-	-	4	10.81	-	-	4	14.29
	Latvia	4	7.02	5	13.51	1	11.11	4	14.29
	Portugal	28	49.12	3	8.11	1	11.11	2	7.14
	Slovenia	4	7.02	3	8.11	1	11.11	2	7.14
	Spain	6	10.53	4	10.81	2	22.22	2	7.14
	Switzerland	-	-	1	2.70	-	-	1	3.57
Years in practice	M	14.98		10.97		11.33		10.85	
	SD	10.50		7.89		8.87		7.72	

**Table 2:** Delphi Round 1 and 2: QoL subdomains, mean ratings with Standard Deviations, and consensus.

	Round 1				Round 2								
					All Participants		Group 1 ^c^		Group 2 ^d^		*t*-Test ^e^		
	M	SD	% Agreement ^a^	CR ^b^	M	SD	M	SD	M	SD	Adj. p ^f^	% Agreement ^g^	CR ^h^
**General domains**													
Physical QoL	6.40	0.8	98.3	*	6.70	0.78	6.89	0.33	6.64	0.87	0.914	94.6	*
Psychological QoL	6.28	0.9	94.7	*	6.70	0.81	6.78	0.67	6.68	0.86	0.931	89.2	*
Social QoL	5.67	1.11	84.2	*	5.81	0.88	5.44	0.73	5.93	0.9	0.799	67.6	-
Spiritual QoL	5.25	1.57	68.4	-	-	-	-	-	-	-	-	-	-
**Physical QoL**													
Functional ability and mobility	6.13	0.88	98.2	*	5.97	0.77	6.11	0.33	5.93	0.87	0.931	88.9	*
Activities of daily living	6.05	0.95	90.9	*	5.94	0.79	6.22	0.67	5.85	0.82	0.914	86.1	*
Fatigue/vitality	6.13	0.92	96.4	*	5.97	0.74	6.33	0.50	5.85	0.77	0.698	91.7	*
Sleep and rest	6.04	0.94	94.6	*	5.94	0.86	5.89	0.78	5.96	0.90	0.931	75.0	*
Pain and discomfort	6.55	0.66	100.0	*	6.81	0.58	6.89	0.33	6.78	0.64	0.931	97.2	*
Health perceptions	5.65	1.17	85.5	*	5.56	0.97	5.56	0.88	5.56	1.01	1.000	58.3	-
Physical symptoms	6.22	0.94	94.6	*	6.14	0.54	6.22	0.44	6.11	0.58	0.931	97.2	*
Health distress	5.91	1.09	90.9	*	5.92	0.84	5.89	0.60	5.93	0.92	0.947	75.0	*
Weight loss/gain	5.69	1.27	85.5	*	5.58	1.20	5.67	1.12	5.56	1.25	0.931	63.9	-
Physical health and comorbidities	5.93	0.88	94.6	*	5.75	1.08	5.78	0.83	5.74	1.16	0.947	72.2	-
**Psychological QoL**													
Anxiety	5.93	1.03	90.9	*	5.91	0.85	5.78	1.09	5.96	0.77	0.931	65.7	-
Depression	6.07	0.88	94.6	*	5.86	0.97	6.11	0.6	5.77	1.07	0.914	85.7	*
Psychological distress	5.85	1.10	83.6	*	6.06	0.80	6.00	0.87	6.08	0.80	0.931	77.1	*
Cognitive functioning, concentration, and attention	5.69	1.09	87.3	*	5.63	0.91	5.22	0.83	5.77	0.91	0.799	57.1	-
Uncertainty	5.31	1.18	76.4	*	5.29	0.99	4.78	0.67	5.46	1.03	0.698	37.1	-
Fear of recurrence	5.73	1.01	90.9	*	5.57	0.95	5.44	0.88	5.62	0.98	0.931	51.4	-
Isolation/abandonment and feelings of belonging	5.73	0.93	89.1	*	5.54	0.92	5.33	0.87	5.62	0.94	0.931	62.9	-
Positive feelings and affect	5.89	0.96	92.7	*	6.14	0.94	6.00	1.00	6.19	0.94	0.931	68.6	-
Negative feelings and affect	5.69	1.07	85.5	*	5.77	1.17	6.00	1.00	5.69	1.23	0.931	71.4	-
Loss of interest in usual activities	5.65	1.00	85.5	*	5.89	0.87	5.78	0.67	5.92	0.93	0.931	68.6	-
**Social QoL**													
Family functioning	5.73	1.03	87.3	*	5.48	0.97	5.33	0.50	5.54	1.10	0.931	51.5	-
Martial functioning	5.40	1.16	83.6	*	5.27	1.07	5.22	0.97	5.29	1.12	0.947	48.5	-
Affection/sexuality	5.27	1.30	72.7	-									
Self-concept/appearance	5.71	1.05	87.3	*	5.82	0.88	6.11	0.93	5.71	0.86	0.914	63.6	-
Enjoyment/leisure	5.78	0.99	90.9	*	5.3	1.21	5.56	1.13	5.21	1.25	0.931	51.5	-
Social activity and limitations	5.69	1.05	85.5	*	5.76	0.83	5.67	0.87	5.79	0.83	0.931	69.7	-
Financial concerns	5.16	1.27	72.7	-									
Social support	5.47	1.09	81.8	*	5.64	1.06	5.89	0.6	5.54	1.18	0.914	60.6	-
Employment	5.47	1.23	80.0	*	5.61	1.00	5.00	1.22	5.83	0.82	0.799	57.6	-
Role limitations due to health or psychical problems	5.76	0.96	87.3	*	5.85	0.76	5.78	0.83	5.88	0.74	0.931	75.8	*
**Spiritual QoL**													
Meaning of illness	5.42	1.32	76.4	*	5.48	0.94	5.56	0.88	5.46	0.98	0.931	48.5	-
Religiosity	4.16	1.73	50.9	-	-	-	-	-	-	-	-	-	-
Hope	5.51	1.41	80.0	*	6.12	1.02	6.22	0.83	6.08	1.10	0.931	75.8	*
Transcendence	4.53	1.59	54.6	-	-	-	-	-	-	-	-	-	-
Inner strength	5.07	1.51	67.3	-	-	-	-	-	-	-	-	-	-

^a^ % of all participants within the top three measures on a 7-point scale. ^b^ * = consensus reached (criterion = 75.0% of the values on the top three measures on a 7-point scale), ^b^ - = consensus not reached. ^c^ Group 1: Participants included in both rounds of the Delphi study. ^d^ Group 2: Participants included only in the 2nd round of the Delphi study. ^e^ Independent samples *t*-test, 2-tailed, unequal variances assumed, *p* adjustment method = BH [45].^f^ Adjusted *p*. ^g^ % of all participants within the top two measures on a 7-point scale. ^h^ * = consensus reached (criterion = 75.0% of the values on top two measures on a 7-point scale), ^h^ - = consensus not reached.

**Table 3:** Mean ranking of the QoL (sub)domains.

Rank	Item	Mean Rank Score ^a^	Consensus Reached (Round 2)
	**General domains**		
1	Physical QoL	1.38	*
2	Psychological QoL	1.76	*
3	Social QoL	2.86	-
	**Physical QoL**		
1	Functional ability and mobility	3.19	*
2	Pain and discomfort	3.42	*
3	Physical symptoms	4.08	*
4	Fatigue/vitality	4.56	*
5	Activities of daily living	4.69	*
6	Physical health and comorbidities	5.83	-
7	Sleep and rest	6.31	*
8	Weight loss/gain	7.61	-
9	Health distress	7.64	*
10	Health perceptions	7.67	-
	**Psychological QoL**		
1	Anxiety	3.14	-
2	Depression	3.37	*
3	Psychological distress	3.89	*
4	Cognitive functioning, concentration, and attention	4.77	-
5	Fear of recurrence	5.31	-
6	Positive feelings and affect	6.34	-
7	Loss of interest in usual activities	6.54	-
8	Isolation/abandonment and feelings of belonging	6.71	-
9	Uncertainty	7.14	-
10	Negative feelings and affect	7.77	-
	**Social QoL**		
1	Family functioning	2.76	-
2	Self-concept/appearance	3.79	-
3	Marital functioning	3.94	-
4	Social activity and limitations	4.00	-
5	Social Support	4.88	-
6	Role limitations due to health or physical problems	5.48	*
7	Enjoyment/leisure	5.55	-
8	Employment	5.61	-
	**Spiritual QoL**		
1	Hope	1.30	*
2	Meaning of illness	1.70	-

^a^ Lower mean score indicates greater importance. * = consensus reached (criterion = 75.0% of the values on the top three measures on a 7-point scale), - = consensus not reached.

## Data Availability

The dataset used and analyzed during the current study is available from the corresponding author upon reasonable request.
